# Genomic diversity, pathogenicity and antimicrobial resistance of *Escherichia coli* isolated from poultry in the southern United States

**DOI:** 10.1186/s12866-022-02721-9

**Published:** 2023-01-16

**Authors:** Aijing Feng, Sadia Akter, Spencer A. Leigh, Hui Wang, G. Todd Pharr, Jeff Evans, Scott L. Branton, Martha Pulido Landinez, Lanny Pace, Xiu-Feng Wan

**Affiliations:** 1 Molecular Microbiology and Immunology, University of Missouri School of Medicine, Columbia, MO USA; 2 Department of Electrical Engineering and Computer Science, University of Missouri, Columbia, MO USA; 3 Christopher S. Bond Life Sciences Center, University of Missouri, Columbia, MO USA; 4 Poultry Research Unit, USDA Agricultural Research Service, Mississippi State, MS USA; 5 Department of Basic Sciences, College of Veterinary Medicine, Mississippi State University, Mississippi State, MS USA; 6 Poultry Research and Diagnostic Laboratory, College of Veterinary Medicine, Mississippi State University, Pearl, MS USA; 7 Mississippi Veterinary Research and Diagnostic Laboratory System, College of Veterinary Medicine, Mississippi State University, Pearl, MS USA

**Keywords:** *Escherichia coli*, Avian Pathogenic *E. coli*., Genomic diversity, Pathogenesis, Antimicrobial resistance, Comparative genomes

## Abstract

*Escherichia coli* (*E. coli*) are typically present as commensal bacteria in the gastro-intestinal tract of most animals including poultry species, but some avian pathogenic *E. coli* (APEC) strains can cause localized and even systematic infections in domestic poultry. Emergence and re-emergence of antimicrobial resistant isolates (AMR) constrain antibiotics usage in poultry production, and development of an effective vaccination program remains one of the primary options in *E. coli* disease prevention and control for domestic poultry. Thus, understanding genetic and pathogenic diversity of the enzootic *E. coli* isolates, particularly APEC, in poultry farms is the key to designing an optimal vaccine candidate and to developing an effective vaccination program. This study explored the genomic and pathogenic diversity among *E. coli* isolates in southern United States poultry. A total of nine isolates were recovered from sick broilers from Mississippi, and one from Georgia, with epidemiological variations among clinical signs, type of housing, and bird age. The genomes of these isolates were sequenced by using both Illumina short-reads and Oxford Nanopore long-reads, and our comparative analyses suggested data from both platforms were highly consistent. The 16 s rRNA based phylogenetic analyses showed that the 10 bacteria strains are genetically closer to each other than those in the public database. However, whole genome analyses showed that these 10 isolates encoded a diverse set of reported virulence and AMR genes, belonging to at least nine O:H serotypes, and are genetically clustered with at least five different groups of *E. coli* isolates reported by other states in the United States. Despite the small sample size, this study suggested that there was a large extent of genomic and serological diversity among *E. coli* isolates in southern United States poultry. A large-scale comprehensive study is needed to understand the overall genomic diversity and the associated virulence, and such a study will be important to develop a broadly protective *E. coli* vaccine.

**Supplementary Information:**

The online version contains supplementary material available at 10.1186/s12866-022-02721-9.

## Introduction

*Escherichia coli* (*E. coli*) are a large and diverse group of bacteria living in the large intestine of human and other warm-blooded animals including the avian species [1, 2]. Although most strains of *E. coli* are not harmful, some of them can be pathogenic and lead to different types of clinical diseases. In chickens, turkeys, and other avian species, Avian Pathogenic *E. coli* (APEC) is responsible for a wide range of localized or systemic extraintestinal-infections commonly called avian colibacillosis, such as colisepticemia, hemorrhagic septicemia, coligranuloma, airsacculitis, swollen‐head syndrome, venereal colibacillosis, coliform cellulitis, peritonitis, salpingitis, orchitis, osteomyelitis/synovitis, panophthalmitis, omphalitis/yolk sac infection, and enteritis [2–6]. APEC is a leading cause of high economic losses in the poultry industry due to decreased productivity, increased mortality, and treatment cost [5, 7–9].

In addition to targeted hygienic and sanitation practices, antibiotics and vaccination are two primary options in reducing the economic losses caused by APEC. However, the usage of antibiotics in agriculture has been associated with the emergence and re-emergence of antibiotic-resistant bacterial strains, causing challenges in microbial prevention and control as well as potential untreatable bacteria posing threats to both human and animal health [10]. Thus, antibiotics are no longer considered a preventative measure, but are restricted to clinical disease treatment. The development of an effective vaccination program continues to be an important strategy in *E. coli* disease prevention and control. Multiple types of *E. coli* vaccines are available, including live attenuated, inactivated, and subunit vaccines, but none have been demonstrated to protect against APEC significantly and consistently [11]. With hundreds of serogroups (may be better than serological diversities) existing among *E. coli* strains [12, 13], a vaccine could be effective against one serogroup but not against those heterologous serogroups. Thus, understanding genetic and phenotypic (particularly antigenicity and pathogenesis) variations of APEC serogroups is important and will facilitate the development of next generation APEC vaccines against APEC-related disease and associated direct and indirect losses.

Conventionally an APEC is defined by pathogenesis in animal experiments, and only a small number of serotypes being identified were associated with APEC [2, 14, 15]. With advances in genomic sequencing technologies in the past two decades, in addition to virulence and serotyping, APEC classification integrates phylogroup and pathogenesis factors derived from genomic analyses [14, 15]. The evolutionary process of bacterial genomes includes mutations, rearrangements, and horizontal transfers. Under certain environmental conditions, bacteria may benefit by acquiring a variable number of accessory and mobile genes that encode adaptive traits through horizontal gene transfers (HGTs), which allow the inheritance of complex phenotype-related characteristics in a single step [16, 17]. Genomic Islands (GEIs) are the outcome of acquiring accessory and mobile genes that form syntenic blocks which insert among closely related strains as discrete DNA segments [18, 19] and play an important role in commensal, symbiotic and environmental bacteria in the evolutionary process for adapting to the prevailing environment [16]. Pathogenicity Islands (PAIs) and Resistance Islands (REIs) are two common GEIs: PAIs carry genes encoding one or more virulence factors [20] and are commonly found in APEC, and REIs carry genes that provide bacterial resistance to antibiotics and commonly found in antimicrobial resistant *E. coli* strains [16, 18, 19]. APEC pathogenesis is characterized by the presence of disease-causing genes (also known as virulence genes) in the PAIs and strains carrying these genes may be responsible for causing colibacillosis [21–23]. Thus, it is imperative to study the presence of PAIs and REIs for understanding the etiology of the disease syndromes in domestic poultry, especially sick birds.

Paired-end sequencing using Illumina MiSeq platform and long read sequencing using MinION sequencer from Oxford Nanopore Technologies (ONT) are two of the primary methods used widely in sequencing microbial genomes [24]. Illumina DNA sequencing platform usually generates more accurate reads than ONT but short reads and can be used for fragmented genome analysis [24, 25]. ONT can be used to produce complete genome analyses. Hybrid method that combing of short and long reads can produce accurate and complete genomes [26]. However, ONT long reads alone were shown to be acceptably accurate when using promising assembly methods [27, 28]. Comparison between the hybrid method and the ONT long reads alone method is needed to justify the feasibility of using long reads alone for a specific study.

In this study, 10 *E. coli* isolates obtained from sick poultry were sequenced using both Illumina MiSeq platform and MinION Oxford Nanopore Technologies, and comparative genomic analyses were performed to understand their association with clinical outcomes by analyzing their genomic diversity, especially the distribution of PAIs and REIs.

## Materials and methods

### Collection of clinical samples

A total of 10 *E. coli* strains were isolated from the representative clinical samples collected from poultry colibacillosis cases submitted for routine diagnostic testing between May 2017 and June 2017. The isolates were from poultry covering diversity in various epidemiological factors such as broiler vs breeder, age, types of localized infection, and types of housing (e.g., commercial vs backyard) (Table 1). Among these samples, nine were collected from Mississippi and one from Georgia. These samples were tested against 18 antibiotics through in-vitro antibiotics resistance analysis (Table 2).

**Table 1:** Epidemiological description of the *E. coli* isolates collected in this study. The multiple samples from different sampling sites of the same bird were pooled for bacteria isolation

Isolate	State	Breed	Age (days)	Specimen	Site of Isolation	Diagnosis
E1	MS	Broiler	4	Live chickens	Yolk sac	*E. coli* yolk sac infection
E2	MS	Backyard chicken	Not informed	Dead chicken	Yolk sac and liver	Severe *E. coli* omphalitis Severe multi-bacterial infection (*E. coli* and *Gallibacterium anatis*)
E3	GA	Broiler	14	Swabs	Air sac	*E. coli* airsacculitis
E4	MS	Pullet	20	Live and dead chickens	Bone marrow, heart and liver	Colisepticemia
E5	MS	Broiler	6	Live and dead chickens	Heart and liver	Colisepticemia
E6	MS	Broiler	4	Live and dead chickens	Heart	Colisepticemia
E7	MS	Broiler	Not informed	Live and dead chickens	Liver	Colisepticemia
E8	MS	Pullet	180	Swab	Hock joint	Multibacterial arthritis (*E. coli* and *Staphylococcus aureus*)
E9	MS	Broiler	5	Live and dead chickens	Yolk sac and liver	*E. coli* yolk sac infection and colisepticemia
E10	MS	Breeder	217	Swab	Air sac	Multi-bacterial airsacculitis (*Escherichia coli* and *Gallibacterium anatis)*

**Table 2:** Antibiotic susceptibility pattern of the *E. coli* isolates collected in this study

Antibiotics	Bacterial Strains									
	Group 1		Group 2		Group 3	Group 4		Group 5		
	E1	E7	E2	E5	E3	E4	E6	E8	E9	E10
Amoxicillin (AMOX)	S^*^	S	S	S	R	R	R	S	S	S
Ceftiofur (TIO)	NI	S	NI	S	NI	R	R	NI	NI	NI
Clindamycin (CLI)	R	R	R	R	R	R	R	R	R	R
Enrofloxacin (ENRO)	S	S	S	S	S	S	S	S	S	S
Erythromycin (ERY)	R	R	R	R	R	R	R	R	R	R
Florfenicol (FFN)	NI	NI	NI	NI	NI	NI	NI	NI	NI	NI
Gentamicin (GEN)	R	R	R	R	R	R	R	S	S	S
Neomycin (NEO)	R	NI	S	S	S	R	R	S	S	S
Novobiocin (NOV)	R	R	R	R	R	R	N/A	R	R	R
Oxytetracycline (OXY)	R	R	NI	NI	NI	R	R	R	NI	NI
Penicillin (PEN)	R	R	R	R	R	R	R	R	R	R
Spectinomycin (SPE)	NI	R	R	R	NI	R	R	R	NI	NI
Streptomycin (STR)	NI	NI	NI	NI	S	NI	NI	NI	NI	S
Sulfathiazole (STZ)	NI	R	R	R	S	R	R	S	S	S
Sulphadimethoxime (SDM)	NI	R	R	R	NI	R	R	I	NI	NI
Tetracycline (TET)	R	R	S	S	S	R	R	R	S	S
Trim/Sulfa (SXT)	S	S	S	S	S	S	S	S	S	S
Tylosin Tartrate (TYLT)	R	R	R	R	R	R	R	R	R	R

^*^: *S* Susceptible, *NI* No Interpretation, *R* Resistant, N/A: data not available

### Bacteria preparation and DNA extraction

All of the *E. coli* isolates were streaked on lysogeny broth [29] agar plates and the plates were incubated at 37 °C overnight. A single colony was picked using a sterile pipet tip and inoculated in 5 ml LB broth in a 15 ml culture tube. The culture tubes were incubated at 37 °C with shaking (150 rpm) overnight using New Brunswick G24 Environmental Incubator shaker. Genomic DNA was extracted using the Qiagen DNeasy Blood and Tissue kit (Qiagen, Germantown, MD), and plasmid by GenJet mini prep kit (Thermo Fisher Scientific, Waltham, MA) as recommended by the manufacturer.

### Genomic sequencing, assembly, and annotation

The genomic sequencing was performed with a 150 bp paired-end run by using Illumina MiSeq platform (Novogene). The quality of Mi-seq reads were checked using FastQC tool (v0.11.8) [30], and Trimmomatic-0.39 was used for trimming the reads with a minimum length of 100 bp and with a minimum phred of 35 [31].

To facilitate genomic assembly, the same DNA samples were also sequenced in house by MinION sequencer from Oxford Nanopore Technologies (ONT). Specifically, 200 ng isolated plasmid DNA was sheered by vortexing, three pulses for at least 10 s each, and then added to 1 µg of genomic DNA. DNA was barcoded and prepared for sequencing using kits EXP-NBD104 and SQK-LSK109 according to manufacturer’s instructions. Sequencing was performed for 48 h using a FLO-MIN106D flow cell and MinKNOW software version 19.05.0 (Oxford Nanopore Technologies, Oxford OX4 4GA, UK). DNA base calling from the Fast5 files and barcode sorting from the resulting Fastq files were performed using Guppy version 5.0.11 and the appropriate sup model (Oxford Nanopore Technologies, Oxford OX4 4GA, UK).

We compared two assembly methods, one using MinION nanopore reads only, and second using a hybrid method combining both Illumina MiSeq reads and MinION nanopore reads. Specifically, MinION nanopore reads were assembled using Flye (v2.9) [27] with ‘–nano-raw –genome-size 5 m –asm-coverage 50’ parameter. Medaka (v1.4.4) was then used for polishing and correcting the MinION assemblies with default parameters [32]. The trimmed Illumina reads were assembled using Unicycler (v0.4.9b) [33] in the short-read-first hybrid assembly mode with the assembled MinION sequences as references using default parameter settings. Mauve (2.5.0) was used to align and compare the assemblies between hybrid assembly and MinION long reads only assembly. Site identity distance was calculated based on the Mauve alignment results. Prokka (v1.14.6) [33] was used for annotating the assemblies with a minimum contig length of 200 bases. Core genome multilocus sequence typing (cgMLST) was conducted using PubMLST [34].

### Genomic data from public database

To explore the genome diversity of the 10 *E. coli* isolates, genomic data for avian *E. coli* isolates (n = 1,463, with the host keyword of ‘chicken’, ‘Gallus gallus’, ‘Gallus gallus domesticus’, ‘poultry’, ‘egg laying hen’, or ‘poultry animal’) were downloaded from the GenBank database. Among these strains, 915 were with genomic annotation, and 12 were labeled as ‘APEC’ in the database.

### Phylogenetic analyses

To identify the evolutionary relationship among (or between) the *E. coli* isolates and those in public databases, genomic analyses using 16 s rRNA and the whole genomic sequences were performed. To avoid multiple sequence alignments among the large number of sequences and make the tree construction be feasible, we use CVTree (v3.0) [35], an alignment free method to compute genetic distances, to construct the composition vector (CV) and the distance matrix among all our tested strains and those from the databases, and then built the phylogenetic trees using the neighbor joining method [36]. Tree visualization was conducted using ggtree R package [37]. Phylogroup of the 10 isolates were identified using ClermonTyping [38].

### Analyses of *E. coli* serotypes

Serotypefinder was used to serotype the isolates by utilizing a reference database containing O-antigen processing system genes *wzx*, *wzy*, *wzm*, and *wzt* for in silico O typing and the flagellin genes *fliC*, *flkA*, *flmA*, *flnA*, and *fllA* for in silico H typing [39].

### Identification of pathogenic and antimicrobial-resistant islands

The annotated results of the 10 isolates were uploaded to a webtool IslandViewer 4 [40] to predict the Genomic Islands (GIs). IslandViewer 4 integrates two sequence composition-based GI prediction methods: IslandPath-DIMOB and SIGI-HMM and one comparative genomics-based GI prediction method: IslandPick. To identify the pathogenic and antimicrobial-resistant islands, the predicted GIs were further mapped with 1) the potential virulence factor (VF) related genes predicted by the virulence genes identifier tool, VFanalyzer [41] and the virulence factor database (VFDB) to label the predicted Pathogenicity Islands (PAI), and 2) the antimicrobial resistance (AMR) genes identified using the Resistance Gene Identifier (RGI) webtool from the Comprehensive Antibiotic Resistance Database (CARD) [42] to label as the predicted REI.

## Results

### In vitro AMR analyses among *E. coli* strains

The 10 isolates were tested against 18 antibiotics through in-vitro AMR analyses, and the AMR pattern showed that all 10 isolates were resistant to four antibiotics, including Penicillin (PEN), Clindamycin (CLI), Erythromycin (ERY), and Tylosin Tartrate (TYLT) and susceptible to both Enrofloxacin (ENRO) and Trim/Sulfa (SXT) (Table 2). The AMR patterns vary greatly among these 10 isolates but they can in general be subdivided into five groups: Group 1 (E1 and E7), Group 2 (E2 and E5), Group 3 (E3), Group 4 (E4 and E6), and Group 5 (E8, E9 and E10). Group 1 is susceptible to Amoxicillin (AMOX) but resistant to Tetracycline (TET) and Gentamicin (GEN); Group 2 susceptible to AMOX and TET but resistant to GEN and Sulfathiazole (STZ); Group 3 susceptible to AMOX, STZ, and TET but resistant to GEN; Group 4 is resistant to all AMOX, GEN, STZ, and TET; Group 5 are susceptible to AMOX, GEN, and STZ but resistant to TET. Of note, E4 and E6 in Group 4 are resistant majority of the testing antibiotics.

### Assembly, annotation, and serotypes among *E. coli* strains

Each of the 10 genomes were assembled by the hybrid method (with Illumina MiSeq and MinION reads) into a single circular chromosome and one or more plasmids. Chromosome sizes, total number of plasmids, plasmid sizes, and the numbers of genomic features (included CDS, CRISPR, gene, rRNA, tRNA, and tmRNA) are shown in Table 3. While the chromosome sizes varied from 4,700,638 to 5,176,723 bases, the total number of plasmids varied from one to six with the plasmid sizes varied from approximately 1 kb to 390 kb. The total number of genes in these isolates varied from 4,599 to 5,406, with CDS ranging from 4,490 to 5,286 and tRNA from 86 to 97. We identified 22 rRNAs and one tmRNA for each isolate.

**Table 3:** Assembly summary from the hybrid method with both Illumina and MinION reads

Isolates	Chromosome size (bp)	Total no. of plasmids	Plasmid size(s) (kb)	CDS	CRISPR	rRNA	tRNA	tmRNA	serotype
E1	4,910,017	5(2*)	390; 50*; 39; 4; 1*	5089	3	22	86	1	O2/O50:H6
E2	5,138,004	6(1*)	124; 103; 83*; 6; 2; 2	5286	2	22	97	1	O123/ O186:H40
E3	4,700,638	2	109; 2	4490	2	22	86	1	O78:H9
E4	4,922,273	4(1*)	146; 119*; 7; 2	4993	1	22	86	1	O7:H4
E5	4,962,527	1	133	4753	2	22	88	1	O No hit: H4
E6	4,876,913	6(1*)	192*;182; 41; 7; 5; 5	5005	2	22	92	1	O84:H20
E7	5,176,723	2	136; 111	5106	2	22	87	1	O78:H4
E8	4,803,012	6(1*)	163; 60; 34; 16*; 4; 1	4799	1	22	88	1	O4:H4
E9	5,118,205	3(1*)	281; 4; 1*	5123	2	22	93	1	O18/O18ac:H49
E10	5,084,019	2	133; 3	4949	1	22	93	1	O2/O50:H4

^*^: incomplete

We compared the assembly derived from the hybrid method with that from the MinION long reads only (Table 4). Chromosome sizes of two assembly methods were very close to each other, and the paired Mauve alignment identity distances for each bacteria were less than 5% between, except for the E8, which had some gaps in the alignment. The numbers of genomic features (included CDS, CRISPR, gene, rRNA, tRNA, and tmRNA) of the hybrid assembly annotation and the annotation of MinION reads only assembly are also close. The same CDS identified in those two types of assemblies were larger than 98%, except for the E8. All the identified serotypes between those two types of assemblies were the same, except for the E8. However, the identified number and size of the plasmids had some difference between those two types of assemblies. The MinION reads only assemblies had more incomplete plasmids, as well as missing some of the plasmids. Overall, the assembly derived from the hybrid method was similar with that from the MinION reads only, particularly at chromosome level.

**Table 4:** Assembly summary from method with the MinION reads only. This table also compares the alignment identity and identified CDS from the hybrid assembly and the assembly with the MinION reads only

Isolates	Chromosome size (bp)	Identity distance compared to hybrid method (%)	Total no. of plasmids	Plasmid size(s) (kb)	CDS	^$^Same identical CDS compared to hybrid method (%)	CRISPR	rRNA	tRNA	tmRNA	serotype
E1	4,909,388	4.14	8 (5*)	380*; 104*; 39; 21*; 3*;1*; 1; 1	5093	98.83	3	22	88	1	O2/O50:H6
E2	5,138,382	2.32	6 (5*)	160*;124; 10*; 5*; 5*; 2*	5964	98.10	2	22	97	1	O123/ O186:H40
E3	4,701,615	0.54	1	109	4951	99.05	2	22	86	1	O78:H9
E4	4,921,781	0.39	3 (2*)	146; 119*; 5*	4990	99.80	1	22	86	1	O7:H4
E5	4,963,620	0.40	1	133	5243	98.93	2	22	89	1	O No hit: H4
E6	4,876,192	3.05	2 (1*)	182; 179*	4950	99.44	2	22	92	1	O84:H20
E7	5,177,108	1.50	2	136; 111	5613	98.84	2	22	87	1	O78:H4
E8	4,816,950	25.85	6 (2*)	155; 111; 68; 61; 11*; 10*	4948	95.85	2	22	91	1	O No hit:H11
E9	5,119,566	1.84	1	281	5959	98.10	2	22	93	1	O18/O18ac:H49
E10	5,083,425	0.33	1	133	4946	99.89	1	22	95	1	O2/O50:H4

^*^: incomplete

^$^: hypothetical proteins were not counted

The O-type among all isolates (except the isolate E5 we could not serotype) were unique, belonging to O2, O4, O7, O18, O18ac, O50, O78, O84, or O123. Six H subtypes were identified: 50% of the isolates are H4 while others are H6, H9, H20, H40 and H49. Except E5 which does not have O:H serotype defined, the rest of the 9 *E. coli* isolates in this study belonged to 9 different O:H groups.

### Pathogenicity Islands of the isolates

**Fig. 1:**
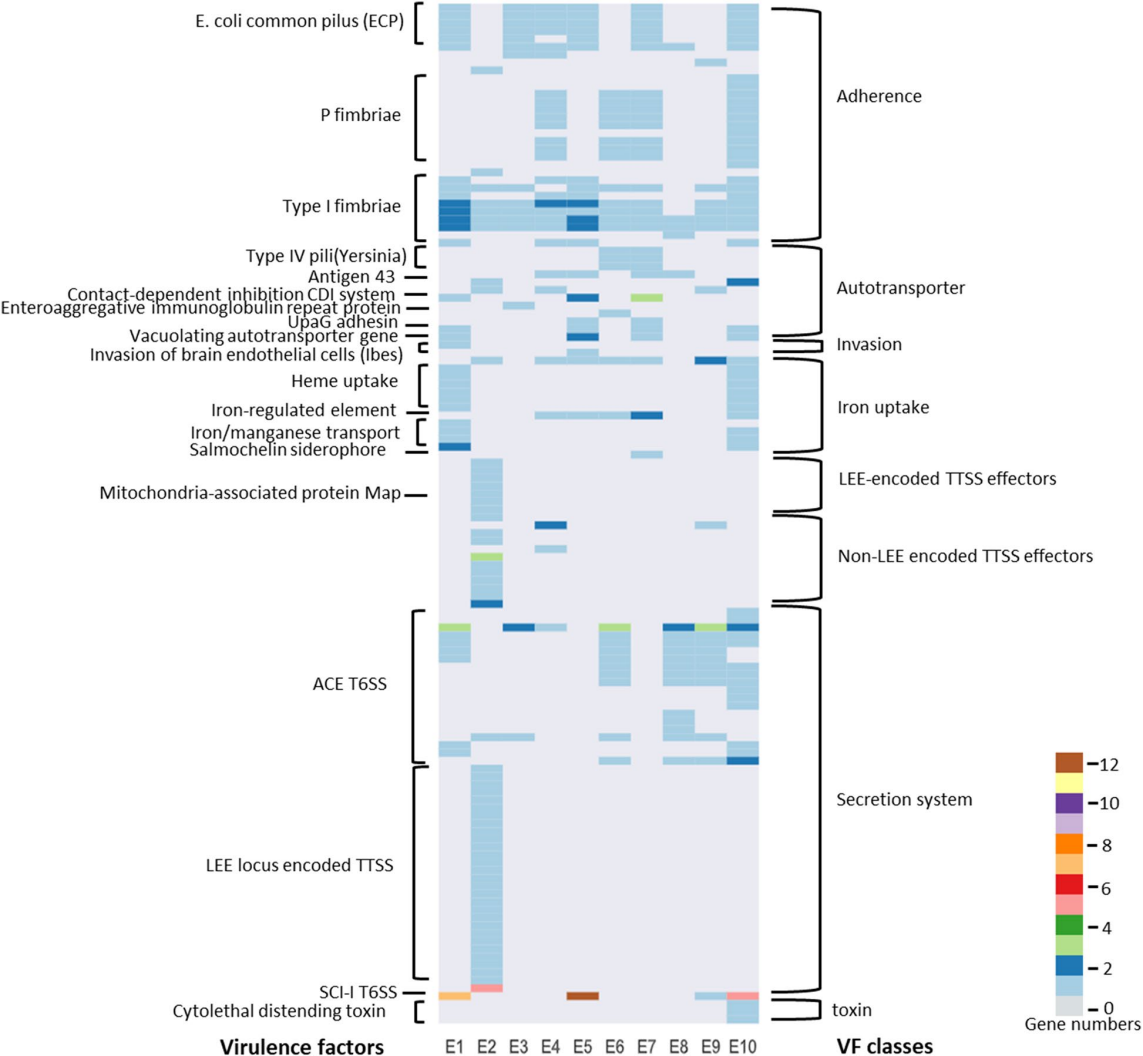
Virulence factors and VF classes identified in the 10 isolates’ PAI. Detailed virulence genes presented in each isolate’ PAI can be found in Table S1

The total number of predicted PAIs in the chromosomes varied from 5 to 16, and the PAI size varied from 4,076 to 101,936 bases. A large set of virulence factors were identified in all 10 isolates, and these virulence genes fall into six VF classes including: (i) adherence, (ii) autotransporter, (iii) invasion, (iv) iron uptake, (v) secretion system, and (vi) toxin (Fig. 1).

Despite the different extent of diversity in PAIs, conserved virulence genes were also identified among these isolates. For example, Type I fimbriae were found in all the 10 isolates. E4, E6 E7 and E10 were found to have P fimbriae related PAIs with a size range from 63.3 to 68.9 kb. The average GC content of the whole genome of each of the 10 isolates was 51% but the average GC contents within these PAIs were: 45% in E10 and 48% in E4, E6 and E7. Each of these four PAIs are located near to the tRNA gene *phe* and includes several virulence genes: one invasion gene (*tia*), several P fimbriae encoded genes (*pap*), one iron-regulated element (*ireA*), multiple insertion sequences and transposase genes. Six other isolates (E1, E3-E5, E7, E10) were found to include *E. coli* common pilus (ECP) in their PAIs. Both E1 and E10 encode a number of genes (n = 10 and 11 respectively) that were associated with iron uptake in the PAI, which is a key mechanism for colonizing the host cells by obtaining iron (a crucial micronutrient) from the host [43]. This includes the heme acquisition system gene *chuA, T, U, W, X,* and *Y* [44]. Also present in these two strains is the *sitB, C, D* iron/manganese transport system [45]. The *sitA* gene is only present in E1. Two other iron related genes were also present; *ireA*, an iron-uptake regulatory element, was found in strains E4, E5, E6, E7, and E10, and *iroD*, a salmochelin siderophore synthesis protein, was found in strain E7 [46, 47]. The presence of these genes in specific strains may enhance their ability to acquire and utilize iron. However, all 10 genomes encode genes for enterobactin production and strains E5, E6, E7, E8, and E10 also encode genes for aerobactin production on a plasmid.

Of interest, the E2 isolate seems to have a very different set of virulence genes from all other isolates described above (Fig. 1). The E2 has a large number of genes that encode a secretion system, specifically, LEE encoded type three secretion system (TTSS) effectors and non-LEE encoded TTSS effectors in its PAI. Among the secretion system associated virulence genes, 45 genes were observed in E2 only compared to 21 unique genes in the other nine isolates. Of note, E2 is an isolate from backyard poultry but all others were from commercial farms.

The E10 isolate was the only one encoding cytolethal distending toxin, which suppresses the proliferation of cells by blocking the eukaryotic cycle at the G2-M transition leading to cell death [48]; and E10 was an isolate from breeders while all other isolates from broilers or pullets.

### AMR determinants of the isolates

**Fig. 2:**
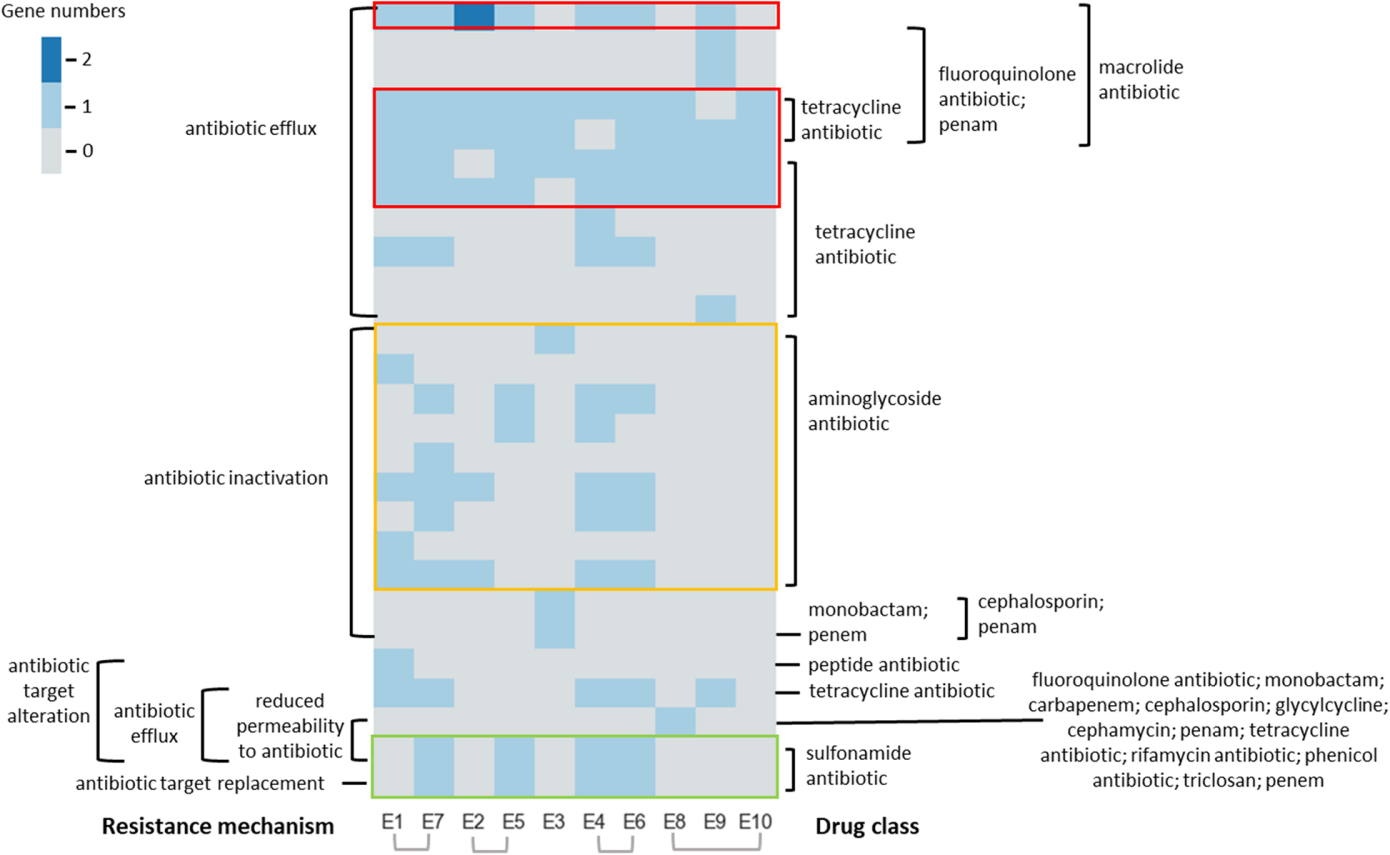
Resistance mechanism and drug class identified in the 10 isolates’ REI. The red boxes indicate the highly prevalent genes of *emrE, evgA, evgS*, *emrK*, and *emrY*. The orange box indicates the genes of *AAC(3)-IId, AAC(3)-IV, AAC(3)-*VIa*, aadA, ANT(3'')-IIa, APH(3'')-Ib, APH(3'')-Ia, APH(4'')-Ia, APH(6'')-Id.* The green box indicates the genes of *sul1* and *sul2*. The order of the genes list in the figures are the same with the above statement. Detailed ARM genes presented in each isolate’s REI can be found in Table S2. The columns were sorted by groups determined in Table 2

*E. coli* isolates in this study have a large diversity in AMR genes (Fig. 2). The REIs of the study isolates contained 26 unique AMR genes and the AMR genes fall into five AMR mechanisms including: (i) antibiotic efflux, (ii) antibiotic inactivation, (iii) antibiotic target alteration, (iv) antibiotic target replacement, and (v) reduced permeability to antibiotic.

Five genes are highly prevalent across the isolates including *emrE*, *emrK*, *emrY*, *evgA*, and *evgS*, whose resistance mechanism is through antibiotic efflux. The *emrE* is a small multidrug resistance (SMR) antibiotic efflux pump having impact on macrolide antibiotic drugs. All four *emrK*, *emrY*, *evgA* and *evgS* genes are major facilitator superfamily (MFS) antibiotic reflux pumps. Both *evgA* and *evgS* genes are resistance-nodulation-cell division (RND)-type efflux pump, both *emrK* and *emrY* genes have impact on tetracycline antibiotic, and both *evgA* and *evgS* genes have impact on macrolide antibiotics, fluoroquinolone antibiotics, penam, and tetracycline antibiotics. Of note, all of E1-E10 are resistant to PEN, CLI, ERY, and TYTL, of which ERY belongs to macrolide antibiotic drugs (Table 2).

The E4, E5, E6, and E7 included *sul1* and *sul2* whereas E2 encodes *sul1* only; the other five isolates had neither *sul1* nor *sul2*. Genes *sul1* and *sul2* that are associated with the resistance mechanism antibiotic target replacement and can impact the sulfonamide antibiotic drug class, which was consistent with the AMR of these five isolates (E2, E4, E5, E6, and E7) to STZ, which is sulfonamide antibiotic (Table 2). The seven isolates of E1-E7 included different genes (*AAC(3)-ID, AAC(3)-IV, AAC(3)-Iva, aadA, ANT(3'')-IIa, APH(3'')-Ib, APH(3'')-Ia, APH(4'')-Ia, APH(6'')-Id*) but all are associated with antibiotic inactivation resistance mechanisms and impact the aminoglycoside antibiotic drug class; this is consistent with AMR pattern showing that all E1-E7 (but not E8-E10) are resistant to GEN, which is an aminoglycoside antibiotic (Table 2).

### Evolutionary analyses

**Fig. 3:**
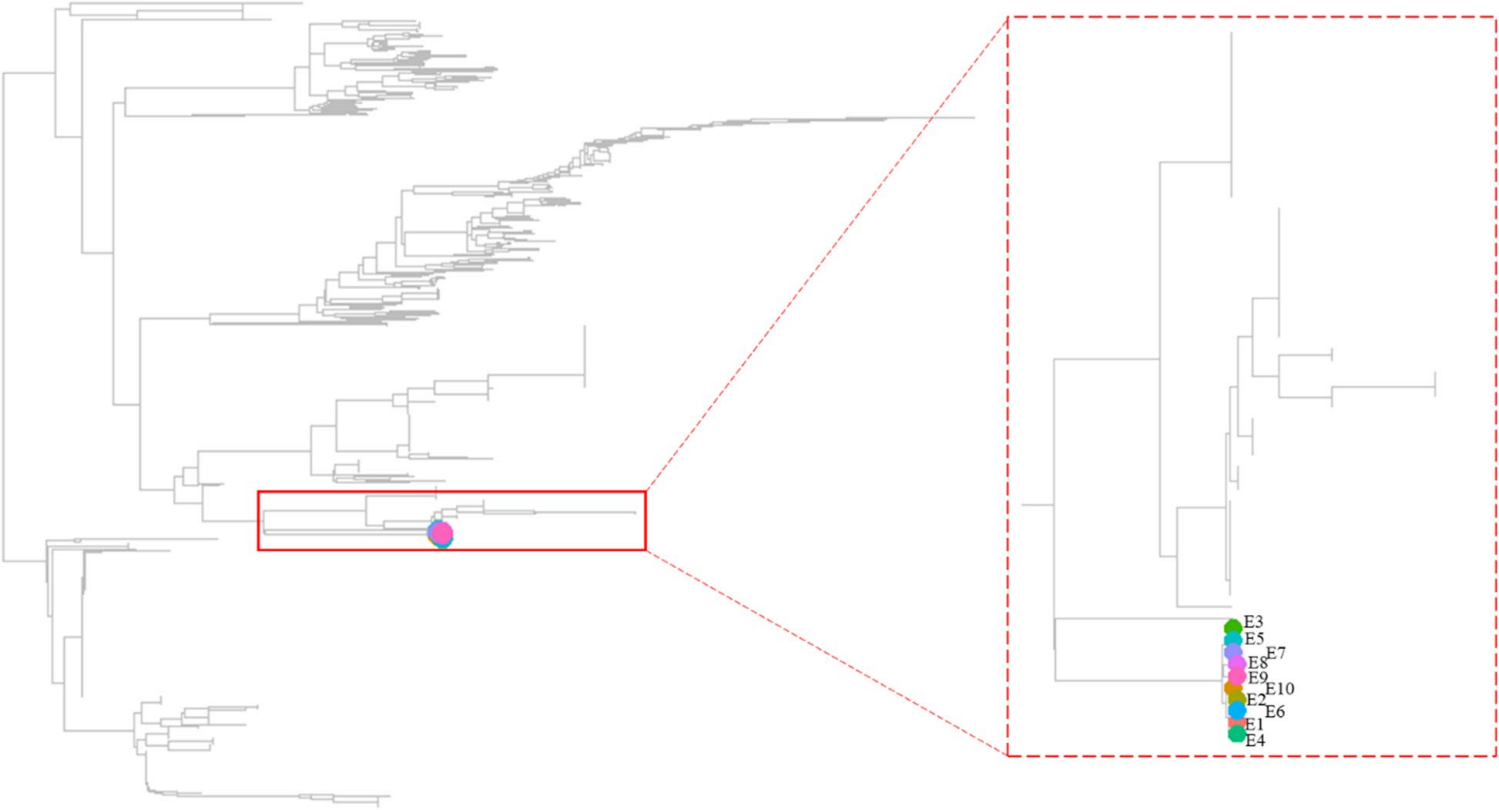
The neighbor joining phylogenetic tree of 16S rRNA for the *E. coli* isolates from southern US poultry. In addition to the 10 *E. coli* isolates we collected for this study, *E. coli* genomes from GenBank (*n* = 1,463) were included in the analyses. Specifically, all 16sRNA from each genome was concatenated, and genetic distances were calculated by using CVTree (v3.0) [35], and the phylogenetic trees were calculated by using neighbor joining method [36]. Tree visualization was conducted using ggtree R package [37]

**Fig. 4:**
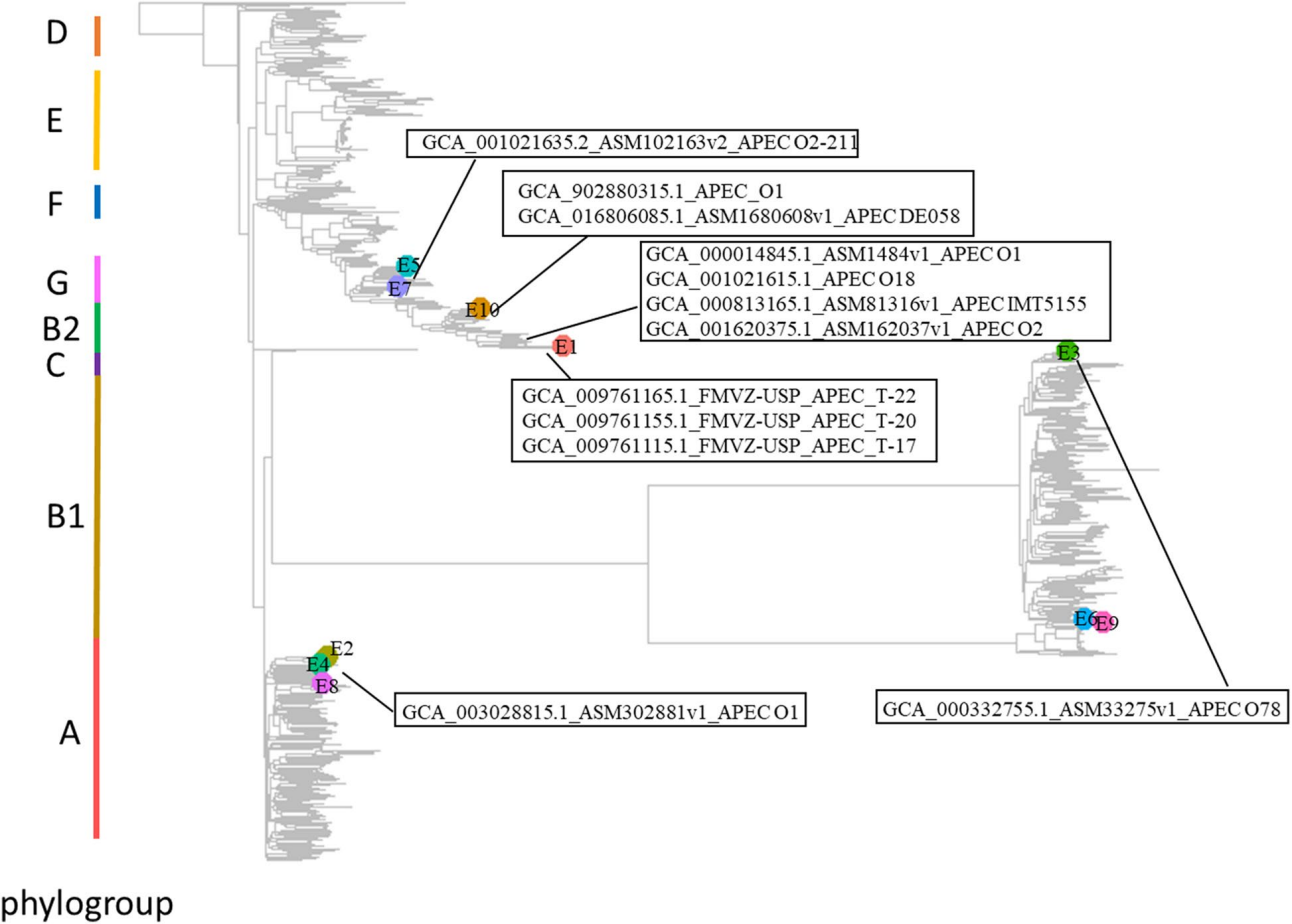
The neighbor joining phylogenetic tree of the whole genome for the *E. coli* isolates from southern US poultry. The boxes mark the APEC representative strains reported in public dataset, which are genetically close to the isolates of this study. Data collection, distance calculation, tree construction and visualization were performed as described in Fig. 3. Color bar in the left of the figure shows the phylogroup cluster of the 10 isolates: E5 and E7 and their close APEC O2-211 belong to the phylogroup G; E10 and E1 and their close 9 APEC belong to the phylogroup B2; E3 and its close APEC O78 belong to the phylogroup C; E6 and E9 belong to the phylogroup B1; E2, E4 and E8 and their close APEC O1 belong to the phylogroup A

To evaluate the genetic diversity and evolutionary relationship of our *E. coli* isolates and those in other public databases (*n* = 1,463), we performed phylogenetic analyses using both 16 s rRNA and genomic sequences. The tree from 16 s rRNA has been conventionally used in defining prokaryotic taxonomy, and the use of a whole genome tree was recently proposed instead [49]. The 16 s rRNA tree showed that all 10 *E. coli* isolates were grouped together in the tree and more genetically similar to each other than those from the public database (Fig. 3). However, whole genome based phylogenetic trees showed that eight genetic groups were formed for all E. coli from public database, and the 12 APEC isolates were identified in phylogroup G, B2, C, and A. Our 10 isolates are distributed across the five major genomic groups of *E. coli* strains (Fig. 4). Of note, compared with most of those *E. coli* from the public databases, our 10 isolates were genetically closer to the APEC isolates reported by other states in the United States.

**Fig. 5:**
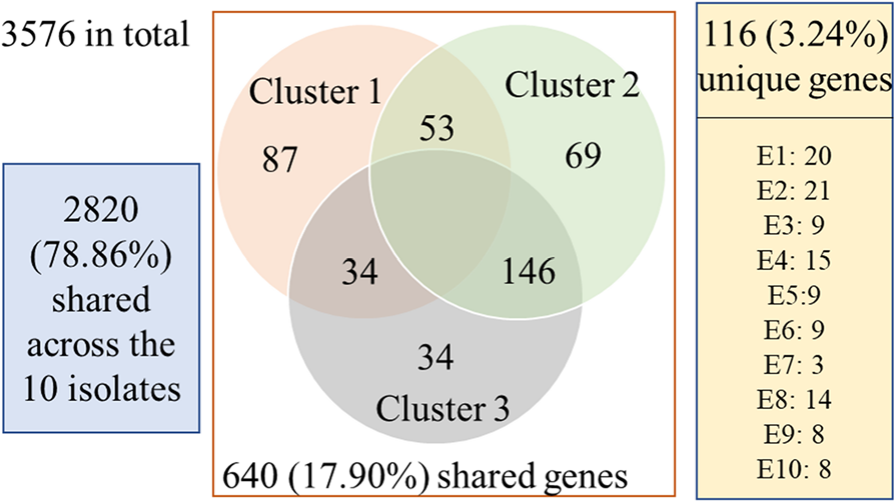
Gene distribution in the 10 isolates. There are 3576 genes with functional annotations (hypothetical proteins were not counted). Among them, 116 (3.24%) genes are unique genes that only present in one of the 10 isolates, while 640 (17.90%) genes were shared with at least two isolates: the cluster 1 (E7, E5, E10 and E1) with 174 shared genes, the cluster 2 (E3, E6, and E9) with 268 shared genes, the cluster 3 (E2, E4 and E8) with 214 shared genes, and 217 genes that shared with at least two isolates but not each entire cluster

**Fig. 6:**
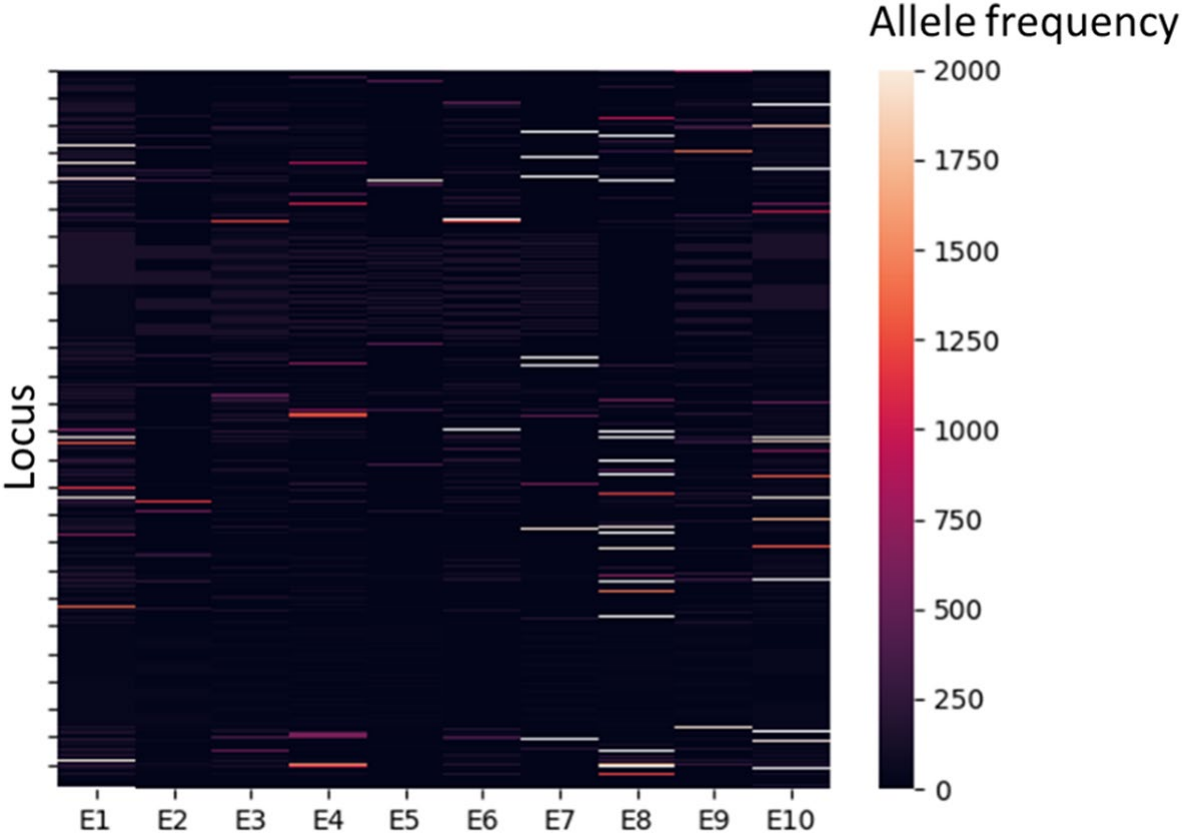
Allele-specific comparisons between the 10 isolates

Comparative analyses suggested that there were overall 3,576 genes with functional annotation (hypothetical proteins were not counted) identified in the 10 isolates. Among these genes, 2,820 (78.86%) genes were shared across these 10 isolates, and the unique genes present in E1-E10 ranged from eight to 21 genes (Fig. 5). On the other hand, 640 (17.90%) genes were shared with at least two isolates: the cluster 1 (E7, E5, E10 and E1) with 174 genes (12 were PAIs, 1 was ARM, 65 were GIs), the cluster 2 (E3, E6, and E9) with 268 genes (2 were PAIs, 64 were GIs), and the cluster 3 (E2, E4 and E8) with 214 genes (1 was PAIs, 1 was ARM, 47 were GIs) (Figs. 4 and 5). Figure 6 shows the allele-specific comparisons between the 10 isolates, and E1, E8 and E10 show large allele frequency than other isolates. Variations within each specific gene need to be further studied.

## Discussion

In this study, we analyzed the genomic diversity of 10 *E. coli* isolates obtained from sick birds from the southern United States, and our results showed a large extent of genomic diversity among these isolates.

Among the 18 tested antibiotics, all of the 10 *E. coli* isolates in this study showed AMR to PEN (penicillins class), CLI (Lincomycins), ERY (macrolides class), and TYTL (tetracyclines class), indicating these isolates were resistant to multiple classes of antibiotics (Table 2). However, there were large variations in AMR patterns for other antibiotics. A large number of reported AMR associated genes were identified, and, in general, the presence of antibiotic class AMR specific genes correlated with the AMR pattern. However, presence of a specific AMR gene may not indicate the AMR phenotype. For example, E10 has a total of 53 AMR associated genes across multiple classes of AMR phenotypes, including 14 genes for AMR against tetracycline; however, E10 is susceptible to tetracycline.

An APEC isolate has been conventionally defined based on the pathogenesis in animal experiment [2, 15], and has been recently mostly integrated data from genomic comparison, particularly those genes related to virulence (e.g. pathogen islands), in addition to the serotyping and clinical diagnosis [14, 15]. Among hundreds of *E. coli* serotypes have been identified, the O78, O18, O1 and O2 are the O-antigen serotypes that most prevalent to APEC [15, 50]. In this study, E1, E3, E6, E7, E9 and E10 belong to these O-antigen serotypes. For the phylogroup, studies showed that B2 and D commonly associated with virulent extra-intestinal infections, and the E1 and E10 in this study belong to B2. Except for B2, the phylogroup of C, G, B1 and A also appeared certain amounts of disease strains [15, 51], and the E2-E9 in this study belong to those phylogroups. Whole genome phylogenic tree shows that the 10 isolates in this study were close to those APEC downloaded from public database. Analysis of the virulence and resistance genes is another important and most used methods for APEC studies [14, 52], including genes of adhesins, invasions, protectins, iron acquisition systems, toxins, and resistant to drug. Those genes were found in our 10 isolates. Introduced by Johnson et al. [53], an isolate can be classified as an Extraintestinal pathogenic Escherichia coli (ExPEC) based on the detection of at least two of the five virulence genes: *papA* and/or *papC* (count as 1), *sfa* and/or *foc* (count as 1), *afa* and/or *dra* (count as 1), *kpsM II*, and *iutA*. All of the 10 isolates in this study have at least two of the ExPEC defining markers. We found *papC* and *iutA* in 100% of our isolates, and *foc* in 90% of the isolates. According to Bonnet et. al. [54] and Mitchell et al. [55], an isolate can be classified as a potential APEC based on the presence of at least four of the five following functional genes or gene groups: (i) *kii*; (ii) *iss*; (iii) *tsh*; (iv) one of the five genes: *sfa, foc, papA, papC*, and *papEF*; and (v) one of the two genes: *iutA* and *fyuA*. The gene/gene group *sfa/foc/papACEF*, *iut/fyuA* and *iss* are highly prevalent among our 10 isolates (100%, 90% and 70%, respectively). Though *kii* and *tsh* are less frequent in our isolates, we found other protectins/serum resistance and toxin genes such as *colV*, *cvaC*, *omp* and *vat*. Thus, all ten isolates were likely to be APEC, and further animal experiments are needed to further confirm the pathogenesis of these isolates.

However, our studies also showed that the list of virulence factors varied among these isolates. In general, the virulence genes among commercial farms, particularly broiler and pullets, were more conserved, despite variations in other epidemiological factors, such as age and location. Many of these conserved virulence genes are associated with adherence activity (i.e., such as P fimbriae, type I fimbriae, and ECP), which allowed the bacteria to adhere to host cells and colonize the body [56–58]. The P fimbriae have been identified as expressed in air sacs of chickens and suggested as possibly participation in the colonization of systemic organs and subsequent septicemia [59]. The fim operon promotes chronic infections by antibiotic evasion [60], and is associated with the intracellular biofilm formation and the urinary tract infections through uropathogenic *E. coli* (UPEC) [61–64]. Type 1 fimbriae are involved in the early stages of the development of colisepticemia [65]. The ECP is an extracellular adhesive fiber that is encoded by the ecpR-E operon [66] and is known as meningitis-associated and temperature-regulated (Mat) fimbria [67]. The ECP genes were reported to be highly prevalent in the *E. coli* isolates from the commercial poultry [68–72]. ECP was used as a candidate antigen for vaccine and elicited an immune response in ExPEC animal disease models including APEC infection in chicken [68, 73, 74].

Isolate E2, originating from backyard chickens, had a high number of secretion system virulence genes unique from other isolates. These genes include LEE loci encoded TTSS and non-LEE encoded by TTSS effectors. The secretion of proteins across phospholipid membranes is a key bacterial virulence strategy including roles in attachment to and intoxication of target cells for scavenging resources and disrupting their functions [75]. Studies have found LEE encoded virulence genes in *E. coli* isolated from poultry and wildlife can lead to attaching and effacing lesions [76]. The LEE encoded TTSS effector genes have also been known to promote enterohemorrhagic *E. coli* (EHEC) pathogenicity [77], and to promote EPEC pathogenicity caused gastroenteritis [78].

The 16S rRNA gene is a highly conserved component of the transcriptional machinery of all DNA-based life forms [79], and minimally affected by horizontal gene transfer [80]. However, variations are still present in certain variable regions, and a previous study showed that, in addition to the these variations, the copy number of 16S rRNA may be different across *E. coli* strains and can be useful for characterizing genome diversity [81]. The copy number of the 16 s rRNA tree in our 10 isolates is 8, and phylogenetic analyses of the concatenated eight 16 s rRNA sequences showed that these 10 isolates were clustered into the same group in the 16 s rRNA tree. Of interest, the whole genome tree showed these isolates were instead clustered into five different groups, each genetically similar to a group of other *E. coli* isolates reported in other states in the United States. Horizontal gene transfer of GIs, which include PAIs, and ARMs, have contributed to the genetic diversity of these isolates and the discrepancy in the topology between the phylogenetic tree derived from 16 s rRNA genes and that from the whole genome for our studied isolates [82]. Incomplete sequencing (not full length) [83] and inaccurate annotation [84] of the 16S rRNA genes had influences in phylogenetic analysis. In this study, the results from both sequencing technologies were consistent. We believe the variation between the tree from whole genome and that from 16 s RNA were likely due to horizontal gene travel events. The functions and the ecological drivers causing these adaptations will need to be further studied.

Sequencing quality of the Illumina reads were higher than the MinION reads for all of the 10 isolates. However, our comparison between the assembly and the annotation between the two different types of reads showed that there were not notable differences in the chromosome assembly and genes identified. However, on plasmid assembly, the hybrid method using both Illumina and MinION reads outperformed the assembly method with the MinION reads only. These results were consistent with the comparison results of *Campylobacter jejuni* sequences assembly using those two methods in the study by Neal-McKenny et al. [24]. Another study showed that even with some challenges, the nanopore sequencing platform is comparable with the Illumina platform in detection of bacterial genera of the nasal microbiota [85]. Those suggest that the nanopore method alone could be an effective method for the genotype analyses of APEC *E. coli*.

## Conclusion

This study explored the genomic diversity of ten *E. coli* isolates obtained from sick birds from the southern US. These isolates were selected from cases with representative colibacillosis disease types. Our analyses showed a large extent of genomic diversity among the studied isolates, including those genetic markers associated with virulence and the presence of AMR associated genes. The presence of virulence and AMR linked genes could vary based on the epidemiological factors such as clinical disease with different signs and lesions, type of housing, and age of the birds. A large-scale comprehensive study is needed to understand the overall genomic diversity and the associated virulence activity and evolution of AMR in Southern US poultry, and such a study will be important to development of a broadly protective *E. coli* vaccine.

## Supplementary Information

**Additional file 1:** **Table S1.** Detailed genes of virulence factors and VF classes identified in the 10 isolates’ PAI. **Table S2.** Detailed ARM genes identified in the 10 isolates’ PAI.

## Data Availability

The genomic datasets used and/or analyzed during the current study are available at the NCBI with the BioProject accession number PRJNA879066 (GenBank BioSample identifier; E1, SAMN30789304; E2, SAMN30789305; E3, SAMN30789306; E4, SAMN30789307; E5, SAMN30789308; E6, SAMN30789309; E7, SAMN30789310; E8, SAMN30789311; E9, SAMN30789312; E10, SAMN30789313).
